# 10 Is Discharge to a Post-Acute Facility Helping or Hurting Our Patients?

**DOI:** 10.1093/jbcr/iraf019.010

**Published:** 2025-04-01

**Authors:** Joya Neal, Ashley Honea, Claudia Islas, Tiffany Hockenberry, Karen Richey, Kevin Foster

**Affiliations:** Diane & Bruce Halle Arizona Burn Center; Diane & Bruce Halle Arizona Burn Center; Diane & Bruce Halle Arizona Burn Center; Diane & Bruce Halle Arizona Burn Center; Diane & Bruce Halle Arizona Burn Center; Diane & Bruce Halle Arizona Burn Center

## Abstract

**Introduction:**

Discharge planning is a critical component of patient care, designed to ensure a smooth transition from the hospital to home or a post-acute care facility (PACF). Prior work at our center has demonstrated that those discharged to a PACF, frequently experience a deterioration of their wound healing, with patients frequently “discharging themselves to home”. The purpose of this study was to identify problems in the discharge process for both patient groups, those discharged directly home and those discharged to PACF.

**Methods:**

This study was conducted in 2 phases. Phase 1 included patients discharged directly from the hospital setting to home. Phase 2 included patients discharged from the hospital to a PACF. Patients were surveyed at their first clinic visit, regarding their discharge experience. Descriptive statistics were calculated for each group. Comparisons between the groups were conducted using chi-square tests for categorical values and Mann-Whitney U tests for continuous variables like age. The analysis focused on identifying significant differences in discharge experiences between the two groups, examining variables such as age, gender, insurance status, issues with medication procurement, understanding of dressing changes, and perception of discharge paperwork.

**Results:**

Patients in Phase 1 had a mean age of 27.88 years, while those in Phase 2 had a significantly higher mean age of 61.02 years (p< 0.001). The gender distribution was not statistically significant between the two phases (p=0.11), however there were more males than females in both groups. There was a significant difference in distribution of insurance status between both phases (p < 0.001). All patients in phase 2 had insurance while phase 1 included both insured and uninsured patients. Phase 2 patients were more likely to report issues with medication procurement after discharge (n=14) compared to phase 1 (n=4, p< 0.01). There was a significant difference in patient understanding of wound care, with Phase 1 patients demonstrating better understanding (p< 0.01). Issues with dressing supply procurement were more frequently reported in Phase 2, though this difference was not statistically significant (p=0.12).

**Conclusions:**

Patients discharged directly to home expressed far greater satisfaction with their experience. This study highlights the distinct challenges faced by patients discharged to post-acute care facilities, particularly with medication procurement and comprehension of wound care. Selection of post-acute care facilities is often dictated by payor source. These facilities often do not have the dressings ordered by the burn center available or they are in limited supply. The findings underscore the need for targeted interventions, particularly for those being discharged to another facility.

**Applicability of Research to Practice:**

Identifying facilities with strong wound care programs and working with payors to facilitate coverage would be a benefit to this population.

**Funding for the Study:**

N/A

